# Identifying Older Adults at Risk of Accelerated Decline in Gait Speed and Grip Strength: Insights from the National Health and Aging Trends Study (NHATS)^†^

**DOI:** 10.3390/jal5020019

**Published:** 2025-06-04

**Authors:** David H. Lynch, Hillary Spangler, Jacob S. Griffin, Anna Kahkoska, Dominic Boccaccio, Wenyi Xie, Feng-Chang Lin, John A. Batsis, Roger A. Fielding

**Affiliations:** 1 Division of Geriatric Medicine, Center for Aging and Health, University of North Carolina at Chapel Hill, Chapel Hill, NC 27599, USA; 2 Cecil G. Sheps Center for Health Services Research, University of North Carolina at Chapel Hill, Chapel Hill, NC 27599, USA; 3 Center for Aging and Health, University of North Carolina at Chapel Hill, Chapel Hill, NC 27599, USA; 4 Department of Anthropology, University of North Carolina at Chapel Hill, Chapel Hill, NC 27599, USA; 5 Department of Nutrition, Gillings School of Global Public Health, University of North Carolina at Chapel Hill, Chapel Hill, NC 27599, USA; 6 Department of Biostatistics, University of North Carolina at Chapel Hill, Chapel Hill, NC 27599, USA; 7 Division of Endocrinology and Metabolism, School of Medicine, University of North Carolina at Chapel Hill, Chapel Hill, NC 27599, USA; 8 Metabolism and Basic Biology of Aging Directive, Jean Mayer USDA Human Nutrition Research Center on Aging, Tufts University, Boston, MA 02111, USA

**Keywords:** gait speed, grip strength, functional decline, older adults, frailty, NHATS

## Abstract

Gait speed and grip strength are widely used measures of physical function in older adults and are predictive of disability, hospitalization, and mortality. However, there is a limited understanding of the long-term trajectories of these measures and which older adults are at the highest risk of functional decline. We used data from the National Health and Aging Trends Study (NHATS) to identify subgroups of community-dwelling older adults with distinct 10-year trajectories in gait speed and grip strength and to examine the baseline factors associated with these patterns. The sample included 4961 adults aged 65 years and older who completed gait speed and grip strength assessments in 2011 and at least one subsequent wave between 2013 and 2021. Using latent class growth analysis, we identified three trajectories for each measure: worsening, stable, and improving. More than one-third of participants were in the worsening group for at least one measure. In multinomial logistic regression models, lower income, Medicaid coverage, cognitive impairment, and multiple chronic conditions were associated with membership in worsening trajectory groups. These findings highlight the heterogeneity of physical aging and the importance of the early identification of older adults who may benefit from targeted interventions to maintain function and independence over time.

## 1. Introduction

Among older adults (≥65 years), functional decline—marked by a reduced ability to perform activities of daily living (ADLs)—is linked to a diminished quality of life and an increased risk of nursing home placement, hospitalization, and mortality [1–4]. Declines in muscle strength and gait speed are particularly significant, as they are strongly associated with ADL impairments [5–9]. Accelerated deterioration in these physical functions closely correlates with higher morbidity and mortality rates [10,11].

Preventing or mitigating declines in muscle strength and gait speed could delay the onset of functional impairments, reduce healthcare burdens, and improve the quality of life for older adults. Stratifying individuals based on their longitudinal trajectories of muscle strength and gait speed offers an opportunity to target interventions toward high-risk groups. However, identifying these subgroups remains challenging due to the multifactorial etiology of functional decline, which integrates demographic, socioeconomic, and clinical factors, complicating efforts to personalize preventative strategies.

In this study, we applied latent class growth analysis (LCGA) to identify subgroups of older adults with distinct trajectories of grip strength and gait speed over a 10-year period. Using a biosocial approach, we also examined the baseline demographic, socioeconomic, and clinical characteristics associated with these functional trajectories to inform targeted interventions. Identifying distinct trajectories of functional change may help target interventions to those at the highest risk of decline and tailor care approaches accordingly. Based on prior research, we anticipated heterogeneity in gait speed and grip strength trajectories among older adults with social determinants of health and comorbidities being important factors in determining functional trajectories.

## 2. Methods

### 2.1. Study Participants

We conducted a secondary analysis of a prospective, longitudinal cohort from the National Health and Aging Trends Study (NHATS), a nationally representative longitudinal cohort of Medicare beneficiaries (n = 8245) aged 65 years and older, collected from 2011 to 2021. Participants were included if they were community-dwelling older adults with at least two measures of walking speed and grip strength. The 2015 replenishment cohort was excluded as recommended, and individuals residing in nursing homes or residential care facilities were excluded due to higher levels of baseline support. The institutional review board from the University of North Carolina at Chapel Hill deemed this study exempt from approval due to the de-identified nature of the data (proof of ethical exemption that was shared with the journal is available upon request–IRB # 23–1085).

### 2.2. Gait Speed and Grip Strength

Walking speed and grip strength were measured at baseline and annually for all participants. For walking speed, participants walked a 3 m course on a level surface, and times were recorded for both the initial walk and the return trip. The use of mobility devices, such as a cane or walker, was allowed and recorded. Individuals using a wheelchair or scooter indoors at all times or unable to walk a short distance were excluded from this test. Grip strength was measured using a dynamometer. Participants were instructed to squeeze the device as hard as possible with their dominant hand, unless they had surgery or a flare-up of pain in both hands or wrists or surgery on both arms or shoulders within the past three months. In such cases, they used their non-dominant hand. Each test was performed twice, and the highest value was recorded. Both assessments followed standardized NHATS protocols. Additional details on instrumentation and reliability are available in NHATS technical documentation.

### 2.3. Covariates

Covariates included self-reported age, race, ethnicity, sex, education level, smoking, marital status, employment status, insurance payer (in addition to Medicare), income, and multiple chronic conditions (MCCs). MCCs were defined as two or more self-reported comorbidities from a list of 10 diseases, including cardiovascular disease, high blood pressure, arthritis, osteoporosis, diabetes, lung disease, stroke, cancer, and dementia.

### 2.4. Statistical Analysis

All data was aggregated into a single dataset.

Participants with at least two measurements of gait speed and grip strength were included (final sample size: 4961). Missing data were imputed using the missForest algorithm, an iterative random forest-based method for mixed-type data.

LCGA was used to model the longitudinal trajectories of gait speed and grip strength changes over time since the first visit. The analysis identified distinct trajectory groups using the highest posterior probability of group membership. The number of classes and functional forms were determined based on model fit criteria (Bayesian Information Criterion, Akaike Information Criterion), interpretability, and group sizes. Solutions with varying numbers of trajectory groups were compared, and the three-group model for each outcome was selected as the most parsimonious, clinically interpretable solution. Group labels (e.g., ‘Worsening’, ‘Stable’, ‘Improving’) were assigned based on the direction and shape of the estimated trajectories over time. Only baseline measurements were included in the model to maintain independence between covariates and outcomes. Demographic and socioeconomic variables and health conditions were compared across trajectory groups using a linear regression model for continuous variables and chi-square tests for categorical variables.

Logistic regression was used to study the association between baseline characteristics and trajectory group membership. We included the following baseline covariates in the logistic regression models to assess their association with the worsening trajectories: age, gender, race, education, income, insurance status, multiple chronic conditions, and smoking status. These variables were selected based on statistical significance (*p* < 0.01) in bivariate analysis.

All analyses were performed using R (version 4.2.2) or SAS software (version 9.4), with adjustments made for the NHATS’s complex survey design through sampling weights.

## 3. Results

The baseline characteristics of the cohort (N = 4961) are shown in Tables 1 and S1–S3. The cohort was predominantly female (55.4%), with the majority aged between 65 and 74 years (43.1%). Comorbid conditions were highly prevalent, with 68.8% reporting multiple chronic conditions. The mean baseline gait speed was 0.85 m/s (SD 0.01), and the mean baseline grip strength was 35.8 kg (SD 0.24) for men and 21.14 kg (SD 0.14) for women.

### 3.1. Latent Class Growth Analysis

**Figure 1.**
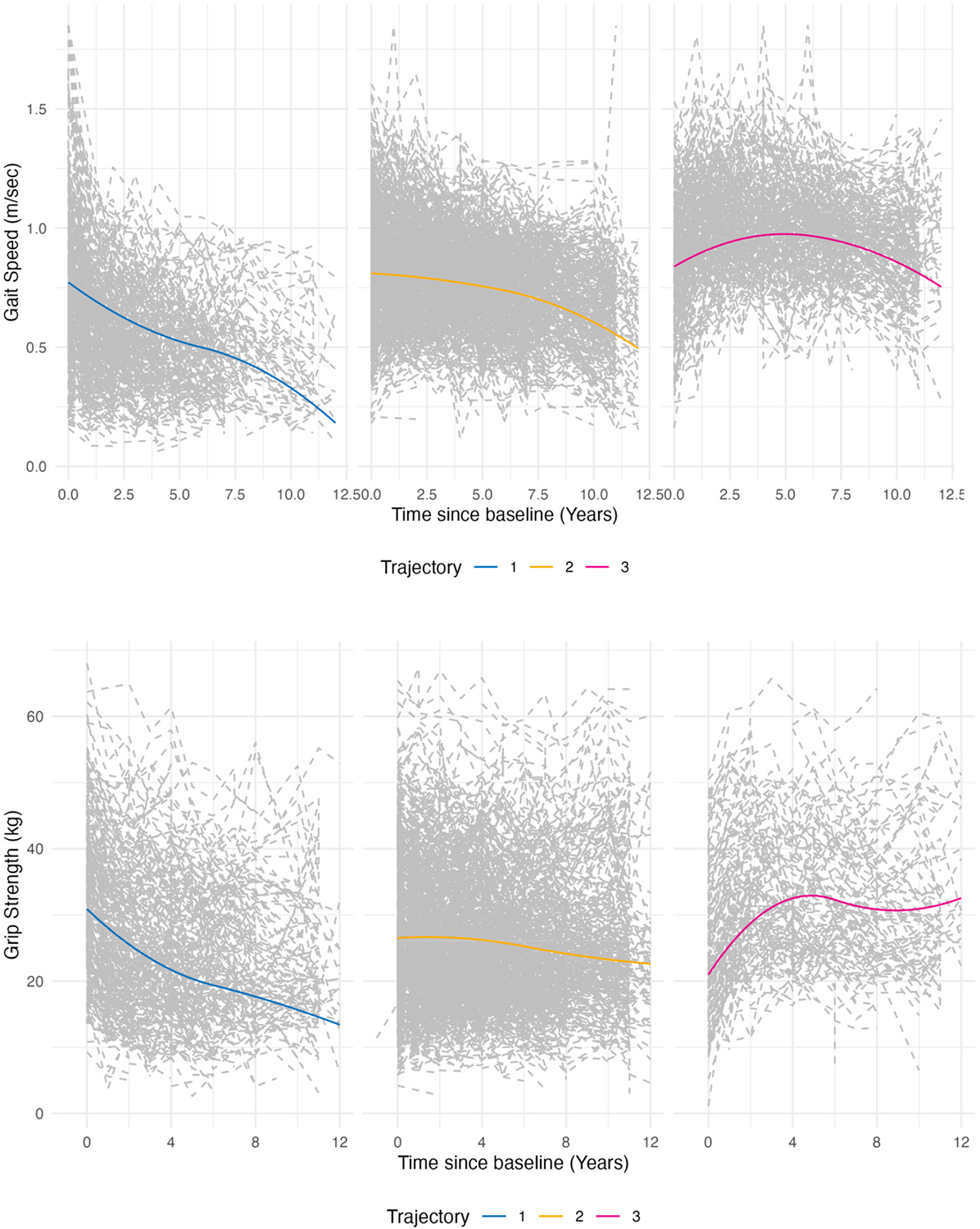
Latent classes identified for gait speed and grip strength. Trajectories of gait speed (**top**) and grip strength (**bottom**) over 10 years. Groups are labeled as 1, 2, and 3 but referred to descriptively in text as ‘Worsening’, ‘Stable’, and ‘Improving’ based on observed patterns.

Latent class growth models identified three distinct trajectory groups for both gait speed and grip strength (Figure 1). For gait speed, the groups were classified clinically as ‘worsening’, ‘stable’, and ‘improving’. The stable group demonstrated a gradual age-related decline in gait speed (*−*0.02 m/s yearly), while the worsening group exhibited a more rapid deterioration in physical function (*−*0.09 m/s yearly). In contrast, the improving group initially showed a slight improvement in gait speed, followed by a mild decline over time (0.03 m/s yearly). Similarly, for grip strength, three distinct trajectory groups were identified: worsening, stable, and improving. The worsening group displayed the steepest decline in grip strength (*−*2.12 kg yearly), while the stable group experienced a more gradual reduction (*−*0.44 kg yearly), and the improving group demonstrated a modest initial improvement followed by a slight decline (1.35 kg yearly).

### 3.2. Baseline Characteristics by Trajectory Classification

Baseline demographic and clinical characteristics differed significantly across trajectory groups (Table 1). Participants in the worsening gait speed group were older, with 30.9% aged ≥85 years compared to 13.2% in the stable group and 3.3% in the improving group (*p* < 0.001; Table 1). The worsening group had a higher proportion of females (61.4%), Black participants (10.5%), and individuals with lower educational attainment, annual income ≤USD 49,999, and greater reliance on public insurance (*p* < 0.001 for all). Additionally, this group had a higher prevalence of cardiovascular disease, arthritis, dementia, and multiple chronic conditions compared to the stable and improving groups (*p* < 0.001 for all). Participants in the worsening grip strength group were older, with 20.0% aged ≥85 years compared to 16.9% in the stable group and 5.6% in the improving group (*p* < 0.001; Table 2). This group had more males (46.3%) compared to the stable (60.4%) and improving (36.7%) groups and a higher prevalence of cardiovascular disease, multiple chronic conditions, and dementia (*p* < 0.001 for all).

### 3.3. Multinomial Logistic Regression Results

Table 2 presents the results of the multinomial logistic regression for worsening gait speed and grip strength trajectories (referent–stable trajectory), showing significant associations with age, education, income, insurance, and multiple chronic conditions. Older adults, especially those aged ≥85 years, had the highest odds of belonging to the worsening trajectory for both gait speed (OR 4.80, 95% CI 3.47–6.64) and grip strength (OR 2.28, 95% CI 1.61–3.21). Higher income was associated with a lower odds of being in the ‘worsening’ trajectory group for gait speed and grip strength. Among those with an income of ≥USD 100,000, the odds ratios were 0.61 (95% CI: 0.41, 0.91) for gait speed and 0.47 (95% CI: 0.30, 0.73) for grip strength. Individuals with private insurance (Medigap or Tricare) had a lower odds of worsening gait speed (OR 0.69, 95% CI 0.51–0.92), but the effect magnitude was smaller for grip strength. Multiple chronic conditions increased the odds of being in the worsening trajectory for both gait speed (OR 1.29, 95% CI 1.07–1.56) and grip strength (OR 1.43, 95% CI 1.21–1.68).

## 4. Discussion

Within this cohort of community-dwelling older adults, our latent class growth analysis (LCGA) identified three distinct trajectories for both gait speed and grip strength: worsening, stable, and improving. This categorization underscores the heterogeneity in the patterns of functional decline among older adults and offers a novel lens to examine changes in physical function over time. By employing a comprehensive biosocial approach, we found that individuals in the worsening trajectory groups for both gait speed and grip strength were disproportionately represented by those aged ≥85 years, with annual incomes ≤USD 49,999, and with multiple chronic conditions at baseline, highlighting the interplay between socioeconomic and clinical factors in driving functional decline. Our findings were generally consistent with expectations and align with prior studies highlighting the influence of sociodemographic and clinical factors on functional decline and emphasizing gait speed as a broader measure of physical performance compared to grip strength in older adults.

Our findings align with prior research emphasizing the critical role of social determinants of health in functional decline, particularly among socioeconomically disadvantaged populations [12–18]. Age, lower income, and a higher burden of chronic conditions were associated with worsening trajectories for both gait speed and grip strength. However, our study extends this understanding by highlighting differences between these measures. Gait speed trajectories were more strongly influenced by socioeconomic factors such as income and insurance status, whereas grip strength trajectories showed weaker associations with these variables. This distinction reflects the broader nature of gait speed as a composite marker of physical function, integrating lower extremity strength, coordination, and balance and capturing the cumulative effects of social and clinical stressors. In contrast, grip strength predominantly reflects upper body strength, which may be less influenced by socioeconomic disparities and stressors over time.

These findings have important implications for researchers and clinicians seeking to address functional decline in aging populations. For researchers, the distinct associations of socioeconomic and clinical factors with gait speed and grip strength suggest that physical function measures should be tailored to specific outcomes and populations. Clinicians can leverage these findings by using tools such as latent class growth analysis (LCGA) to classify patients into distinct functional trajectories. The early identification of patients in the worsening trajectory groups, particularly for gait speed, could inform targeted interventions. These might include resistance training programs, mobility aids, and comprehensive care plans that address both health and socioeconomic challenges [19]. Gait speed, in particular, emerges as a robust, longitudinal measure of physical function and may be more reliable for monitoring changes over time compared to grip strength, which can fluctuate due to short-term factors.

Future research should focus on two complementary pathways. First, epidemiological studies are needed to further explore the dynamic interplay between socioeconomic, clinical, and environmental factors that influence functional trajectories. This could include refining risk prediction models, integrating a broader range of biosocial variables, and examining the pathways through which socioeconomic disparities impact functional outcomes. Second, intervention studies are critical to testing strategies that mitigate functional decline in high-risk groups. Programs that combine social support, chronic disease management, and physical activity tailored to individual needs should be rigorously evaluated. Examples of relevant supports may include transportation services, in-home caregiving, and connections to community-based aging resources. Expanding access to preventive services, such as physical therapy and exercise programs, through public insurance coverage and community-based initiatives, will be essential for addressing disparities in functional outcomes and improving the quality of life for older adults. For individuals in the worsening trajectory group, more accessible options such as home-based physical activity programs or caregiver-supported mobility interventions may be better suited than traditional exercise programs.

Our study has several strengths. Using a large, nationally representative NHATS sample enhanced the generalizability of our findings to community-dwelling older adults. The longitudinal design and latent class growth analysis (LCGA) provided a nuanced understanding of functional trajectories over a decade, capturing heterogeneity in physical function. Logistic regression further identified key baseline factors, such as age, income, and chronic conditions, associated with worsening trajectories. However, several limitations should be acknowledged. The observational nature of this study limits causal inference. While LCGA is a valuable tool for classifying trajectories, its performance depends on data quality, and missing data, despite imputation, may have introduced bias. We did not adjust for multiple comparisons, and the results should be interpreted in the context of potential Type I error. We considered potential overlap among sociodemographic variables and selected covariates based on conceptual relevance and model interpretability. Additionally, short-term variability in gait speed and grip strength assessments reduces their utility as stable longitudinal measures. Future studies should incorporate additional physical function measures to provide a more comprehensive assessment.

These findings underscore the critical role of social determinants of health—such as income, insurance status, and educational attainment—in shaping functional trajectories, particularly for gait speed. Using a comprehensive biosocial approach and latent class growth analysis (LCGA), this study highlights gait speed as a sensitive longitudinal marker that captures the cumulative impact of socioeconomic and clinical factors. These results emphasize the need for targeted interventions addressing both medical and social determinants of functional decline, including chronic disease management and social support. Future research should validate these findings in diverse populations and develop scalable interventions to mitigate disparities and improve outcomes in aging populations.

## Supplementary Material

Supplement

**Supplementary Materials:** The following are available online at https://www.mdpi.com/article/10.3390/jal5020019/s1, Supplemental Table S1: Baseline characteristics of participants by grip strength trajectory classification. Supplemental Table S2: Baseline characteristics of demographic information for older adults stratified by baseline gait speed quartiles among 2011 cohort. Supplemental Table S3: Baseline characteristics of demographic information for older adults stratified by baseline grip strength quartiles among 2011 cohort.

## Figures and Tables

**Table 1.** Baseline characteristics of participants by gait speed trajectory classification.

Variable—Mean (SD)	Total (N = 4961)	Worsening (N = 1409)	Stable (N = 2709)	Improving (N = 843)	*p*-Value
Baseline Gait Speed	0.85 (0.01)	0.79 (0.02)	0.86 (0.01)	0.91 (0.01)	<0.001
Yearly Change in Gait Speed	−0.03 (0.002)	−0.09 (0.004)	−0.02 (0.002)	0.03 (0.004)	<0.001
		Demographics—N (%)			
Age 65–74 years	2140 (43.1)	355 (25.2)	1194 (44.1)	591 (70.1)	<0.001
75–84 years	1999 (40.3)	618 (43.9)	1157 (42.7)	224 (26.6)	
≥85 years	822 (16.6)	436 (30.9)	358 (13.2)	28 (3.3)	
Gender: Female	2822 (55.4)	891 (61.4)	1527 (56.0)	404 (47.7)	<0.001
Race White	3553 (82.8)	923 (77.8)	1962 (82.9)	668 (87.7)	<0.001
Black	980 (7.3)	351 (10.5)	516 (7.0)	113 (4.7)	
Hispanic	269 (6.1)	81 (7.1)	152 (6.4)	36 (4.3)	
Other	159 (3.8)	54 (4.5)	79 (3.6)	26 (3.3)	
Marital Status Married/Living with Partner	2627 (53.0)	572 (40.6)	1466 (54.1)	589 (69.9)	<0.001
Non-partnered	2334 (47.0)	837 (59.4)	1243 (45.9)	254 (30.1)	
		Socioeconomic Status			
Education High School or Less	2505 (50.5)	855 (60.7)	1348 (49.8)	302 (35.8)	<0.001
Some College or More	2456 (49.5)	554 (39.3)	1361 (50.2)	541 (64.2)	
Income Low (≤USD 49,999)	2032 (41.0)	641 (45.5)	1139 (42.0)	252 (29.9)	<0.001
Middle (USD 50,000–99,999)	622 (12.5)	104 (7.4)	364 (13.4)	154 (18.3)	
High (≥USD 100,000)	296 (6.0)	38 (2.7)	149 (5.5)	109 (12.9)	
Missing	2011 (40.5)	626 (44.4)	1057 (39.0)	328 (38.9)	
Insurance Public Insurance	2116 (42.7)	658 (46.7)	1135 (41.9)	323 (38.3)	<0.001
Private Insurance	2845 (57.3)	751 (53.3)	1574 (58.1)	520 (61.7)	
		Health Conditions			
Arthritis	2636 (53.1)	856 (60.8)	1406 (51.9)	374 (44.4)	<0.001
Cancer	1272 (25.6)	366 (26.0)	706 (26.1)	200 (23.7)	0.21
Cardiovascular Disease	1208 (24.3)	435 (30.9)	635 (23.4)	138 (16.4)	<0.001
Current Smoker	413 (8.3)	104 (7.4)	244 (9.0)	65 (7.7)	0.02
Dementia	127 (2.6)	75 (5.3)	44 (1.6)	8 (0.9)	<0.001
Elevated Waist Circumference	3070 (61.9)	952 (67.6)	1710 (63.1)	408 (48.4)	<0.001
High Blood Pressure	3275 (66.0)	982 (69.7)	1822 (67.3)	471 (55.9)	<0.001
Low Physical Activity	1381 (27.8)	511 (36.3)	718 (26.5)	152 (18.0)	<0.001
Lung Disease	703 (14.2)	226 (16.0)	382 (14.1)	95 (11.3)	0.04
Osteoporosis	972 (19.6)	320 (22.7)	511 (18.9)	141 (16.7)	<0.001
Type II Diabetes	1174 (23.7)	399 (28.3)	639 (23.6)	136 (16.1)	<0.001
Unintentional Weight Loss	638 (12.9)	260 (18.4)	307 (11.3)	71 (8.4)	<0.001
Multiple Chronic Conditions (≥2)	3554 (71.6)	1110 (78.8)	1947 (71.9)	497 (58.9)	<0.001

Percentages reflect the distribution within each trajectory group unless otherwise noted. Marital Status: ‘Married/Living with Partner’ includes individuals who are either married or living with a partner. ‘Non-partnered’ includes those who are never married, divorced, separated, or widowed. Income: ‘Low’ includes individuals with an income ≤USD 49,999, ‘Middle’ includes USD 50,000–USD 99,999, and ‘High’ includes ≥USD 100,000. The ‘Missing’ category includes individuals who did not provide income information. Insurance: ‘Public Insurance’ includes Medicaid and Medicare only, and ‘Private Insurance’ includes Medigap and Tricare. Health Conditions: ‘Multiple chronic conditions’ refers to individuals with two or more chronic conditions. ‘Elevated Waist Circumference’ is defined as a waist circumference ≥102 cm for men and ≥88 cm for women. Statistical Testing: *p*-values are calculated using chi-square tests for categorical variables and ANOVA for continuous variables.

**Table 2.** Multinomial logistic regression for worsening gait speed and grip strength trajectories.

Variable	Worsening Gait Speed OR (95% CI) (Ref: Stable)	Worsening Grip Strength OR (95% CI) (Ref: Stable)
	Demographics	
Age		
65–74 years	Reference	Reference
75–84 years	1.74 (1.33, 2.27)	1.33 (1.03, 1.73)
≥85 years	4.80 (3.47, 6.64)	2.28 (1.61, 3.21)
Race		
White	Reference	Reference
Black	1.43 (1.11, 1.85)	0.98 (0.79, 1.22)
Hispanic/Other	1.10 (0.84, 1.45)	1.18 (0.83, 1.58)
Gender		
Male	Reference	Reference
Female	1.08 (0.91, 1.29)	1.55 (1.15, 2.08)
	Socioeconomic Status	
Education		
High School or Less	Reference	Reference
Some College	0.67 (0.53, 0.85)	1.03 (0.78, 1.36)
Marital Status		
Married/Living with Partner	Reference	Reference
Non-partnered	1.10 (0.84, 1.41)	0.94 (0.72, 1.22)
Income		
Low (≤USD 49,999)	Reference	Reference
Middle (USD 50,000–99,999)	0.68 (0.53, 0.88)	0.60 (0.43, 0.84)
High (≥USD 100,000)	0.61 (0.41, 0.91)	0.47 (0.30, 0.73)
Insurance		
Public Insurance	Reference	Reference
Private Insurance	0.69 (0.51, 0.92)	0.84 (0.61, 1.15)
	Health Conditions	
Multiple Chronic Conditions (≥2)	1.29 (1.07, 1.56)	1.43 (1.21, 1.68)

## Data Availability

Please see https://www.nhats.org/researcher/nhats (accessed on 22 May 2025) for information on accessing all data presented in this paper.
