# Supplementary material for: Identifying Older Adults at Risk of Accelerated Decline in Gait Speed and Grip Strength: Insights from the National Health and Aging Trends Study (NHATS)
Source: J Ageing Longev. Author manuscript; Available in PMC 2026 Feb 25. (PMC12931739; doi:10.3390/jal5020019)
Supplement: Supplement [file NIHMS2147500-supplement-Supplement.zip › jal-3575772-supplementary.pdf]

Supplementary material:

Table S1. Baseline characteristics of participants by grip strength trajectory classification.

Variable—Mean (SD)	Total (N = 4961)	Worsening (N = 1015)	Stable (N = 3521)	Improving (N = 425)	*p*-Value
Baseline Grip Strength	27.68 (0.20)	31.53 (0.52)	26.51 (0.25)	28.44 (0.72)	<0.001
Female	21.14 (0.14)	38.60 (0.52)	35.13 (0.32)	33.99 (0.71)	<0.001
Male	35.80 (0.24)	23.32 (0.34)	20.86 (0.16)	18.86 (0.25)	
Yearly Change in Grip Strength	−0.60 (0.04)	−2.12 (0.11)	−0.44 (0.04)	1.34 (0.24)	<0.001
Demographics—N (%)					
Age<br>65–74 years	2140 (43.1)	391 (38.5)	1475 (41.9)	274 (64.5)	<0.001
75–84 years	1999 (40.3)	421 (41.5)	1451 (41.2)	127 (29.9)	
≥85 years	822 (16.6)	203 (20.0)	595 (16.9)	24 (5.6)	
Gender: Female	2822 (55.4)	480 (46.3)	2173 (60.4)	169 (36.7)	
Race<br>White	3553 (82.8)	707 (82.2)	2567 (83.2)	279 (81.6)	0.03
Black	980 (7.3)	210 (7.3)	657 (6.9)	113 (10.6)	
Hispanic	269 (6.1)	58 (5.9)	189 (6.2)	22 (5.5)	
Other	159 (3.8)	40 (4.6)	108 (3.7)	11 (2.4)	
Marital Status<br>Married/Living with Partner	2627 (53.0)	552 (54.4)	1793 (50.9)	282 (66.4)	<0.001
Non-partnered	2334 (47.0)	463 (45.6)	1728 (49.1)	143 (33.6)	
Socioeconomic Status					
Education<br>High School or Less	2505 (50.5)	519 (51.1)	1787 (50.8)	199 (46.8)	0.03
Some College or More	2456 (49.5)	496 (48.9)	1734 (49.2)	226 (53.2)	
Income<br>Low ≤ USD 49,999)	2032 (41.0)	444 (43.7)	1413 (40.1)	175 (41.2)	0.12
Middle (USD 50,000–99,999)	622 (12.5)	111 (10.9)	454 (12.9)	57 (13.4)	
High (≥USD 100,000)	296 (6.0)	52 (5.1)	211 (6.0)	33 (7.8)	
Missing	2011 (40.5)	408 (40.2)	1443 (41.0)	160 (37.6)	
Insurance<br>Public	2116 (42.7)	449 (44.2)	1471 (41.8)	196 (46.1)	0.57
Private	2845 (57.3)	566 (55.8)	2050 (58.2)	229 (53.9)	0.57
Health Conditions					
Cancer	1272 (25.7)	280 (28.4)	880 (24.5)	112 (28.5)	0.05
Arthritis	2636 (53.1)	560 (55.2)	1853 (52.6)	223 (52.5)	0.24
Cancer	1272 (25.6)	280 (27.6)	880 (25.0)	112 (26.4)	0.05
Cardiovascular Disease	1208 (24.3)	250 (24.6)	851 (24.2)	107 (25.2)	0.99
Current Smoker	413 (8.3)	73 (7.2)	295 (8.4)	45 (10.6)	0.05
Dementia	127 (2.6)	43 (4.2)	81 (2.3)	3 (0.7)	<0.001
Elevated Waist Circumference	3070 (61.9)	602 (59.3)	2211 (62.8)	257 (60.5)	0.57
High Blood Pressure	3275 (66.0)	694 (68.4)	2306 (65.5)	275 (64.7)	0.002
Low Physical Activity	1381 (27.8)	280 (27.6)	993 (28.2)	108 (25.4)	0.38
Lung Disease	703 (14.2)	148 (14.6)	491 (13.9)	64 (15.1)	0.43
Multiple Chronic Conditions	3554 (71.6)	751 (74.0)	2497 (70.9)	306 (72.0)	0.04

Osteoporosis	972 (19.6)	181 (17.8)	718 (20.4)	73 (17.2)	0.19
Type II Diabetes	1174 (23.7)	249 (24.5)	801 (22.7)	124 (29.2)	0.19
Unintentional Weight Loss	638 (12.9)	126 (12.4)	456 (13.0)	56 (13.2)	0.93

Marital Status: ‘Married/Living with Partner’ includes individuals who are either married or living with a partner. ‘Non-partnered’ includes those who were never married or are divorced, separated, or widowed. Income: ‘Low’ includes individuals with an income ≤ USD 49,999, ‘Middle’ includes USD 50,000–USD 99,999, and ‘High’ includes ≥ USD 100,000. The ‘Missing’ category includes individuals who did not provide income information. Insurance: ‘Public Insurance’ includes Medicaid and Medicare only, and ‘Private Insurance’ includes Medigap and Tricare. Health Conditions: ‘Multiple chronic conditions’ refers to individuals with two or more chronic conditions. ‘Elevated Waist Circumference’ is defined as a waist circumference ≥ 102 cm for men and ≥88 cm for women. Statistical Testing: p-values are calculated using chi-square tests for categorical variables and ANOVA for continuous variables.

Table S2. Baseline characteristics of demographic information for older adults stratified by baseline gait speed quartiles among 2011 cohort.

	**Total (N = 4961)**	**Q1 (N = 1138)**	**Q2 (N = 1523)**	**Q3 (N = 1123)**	**Q4 (N = 1177)**	
**Variable**	**N (%) Mean (SE)**	**N (%) Mean (SE)**	**N (%) Mean (SE)**	**N (%) Mean (SE)**	**N (%) Mean (SE)**	***p*-Value**
Yearly Change in Gait Speed	−0.03 (0.00)	0.01 (0.01)	−0.01 (0.00)	−0.03 (0.00)	−0.06 (0.00)	<0.001
Demographics<br>Age<br>65–69 years	1026 (30.3)	103 (13.7)	249 (24.2)	274 (33.1)	400 (43.6)	<0.001
70–74 years	1114 (26.2)	176 (19.9)	309 (23.9)	289 (28.8)	340 (29.9)	
75–79 years	1043 (19.4)	243 (22.7)	338 (21.4)	252 (20.4)	210 (14.8)	
80–84 years	956 (13.8)	260 (19.3)	344 (17.9)	197 (11.9)	155 (8.3)	
85+ years	822 (10.3)	356 (24.3)	283 (12.6)	111 (5.7)	72 (3.4)	
Gender<br>Male	2139 (44.6)	490 (44.2)	646 (44.0)	487 (45.3)	516 (44.9)	0.94
Female	2822 (55.4)	648 (55.8)	877 (56.0)	636 (54.7)	661 (55.1)	
Race<br>White	3553 (82.8)	685 (72.6)	1015 (78.9)	862 (86.1)	991 (89.9)	<0.001
Black	980 (7.3)	323 (12.4)	360 (9.5)	185 (6.2)	112 (3.2)	
Hispanic	269 (6.1)	97 (11.2)	83 (6.5)	48 (4.8)	41 (3.8)	
Other	159 (3.8)	33 (3.9)	65 (5.1)	28 (3.0)	33 (3.1)	
Education<br><High School	1149 (18.7)	417 (33.5)	417 (23.6)	180 (13.9)	135 (9.2)	<0.001
High School	1344 (26.7)	327 (29.7)	438 (30.2)	331 (29.4)	248 (19.6)	
Some College	663 (14.4)	120 (11.1)	190 (13.5)	160 (15.0)	193 (16.7)	
≥College Degree	1782 (40.2)	273 (25.7)	461 (32.8)	450 (41.7)	598 (54.5)	
Marital Status<br>Married	2518 (56.8)	442 (43.4)	706 (50.6)	623 (60.5)	747 (67.7)	<0.001
Separated/Divorced	608 (12.2)	136 (12.2)	208 (14.7)	141 (11.5)	123 (10.5)	
Widowed	1547 (25.2)	493 (39.0)	516 (28.8)	295 (21.7)	243 (16.4)	
Never Married	175 (3.3)	50 (3.8)	57 (3.6)	32 (3.2)	36 (2.9)	
Living with Partner	108 (2.5)	17 (1.6)	31 (2.3)	32 (3.2)	28 (2.5)	

Residence Type<br>Community	4762 (96.1)	1062 (92.3)	1455 (95.2)	1089 (97.1)	1156 (98.5)	<0.001
Residential Care	199 (3.9)	76 (7.7)	68 (4.8)	34 (2.9)	21 (1.5)	
Income<br>USD 0–24,999	1314 (23.5)	420 (35.3)	455 (28.6)	261 (21.3)	178 (13.6)	<0.001
USD 25,000–49,999	718 (14.8)	123 (11.8)	211 (14.2)	186 (16.2)	198 (16.0)	
USD 50,000–74,999	411 (9.3)	54 (5.1)	102 (7.4)	125 (11.8)	130 (11.7)	
USD 75,000–99,999	211 (5.4)	24 (2.1)	45 (3.8)	70 (7.8)	72 (6.9)	
USD 100,000–199,999	219 (5.8)	28 (2.9)	36 (3.0)	60 (6.8)	95 (9.5)	
USD 200,000+	77 (2.1)	5 (0.6)	14 (1.3)	20 (2.0)	38 (3.8)	
MISSING	2011 (39.0)	484 (42.2)	660 (41.8)	401 (34.1)	466 (38.5)	
Language<br>Spanish	717 (16.4)	165 (17.9)	219 (16.6)	140 (13.7)	193 (17.7)	0.07
English	4091 (83.6)	912 (82.1)	1245 (83.4)	966 (86.3)	968 (82.3)	
Insurance<br>Medicaid	610 (9.5)	234 (17.7)	220 (12.8)	96 (6.9)	60 (3.8)	<0.001
Medigap	2689 (56.7)	525 (49.0)	804 (54.6)	644 (58.9)	716 (61.4)	
Tricare	156 (3.3)	35 (3.4)	34 (2.3)	40 (3.6)	47 (4.0)	
Medicare only	1506 (30.4)	344 (29.8)	465 (30.3)	343 (30.6)	354 (30.7)	
Health<br>Current Smoker	381 (15.3)	91 (18.0)	126 (15.9)	91 (16.1)	73 (12.3)	0.08
Cardiovascular Disease	1208 (22.7)	348 (30.4)	385 (24.2)	257 (21.3)	218 (18.0)	<0.001
Comorbidities<br>Arthritis	2630 (51.5)	699 (61.5)	865 (56.6)	573 (51.2)	493 (41.0)	<0.001
Osteoporosis	972 (20.0)	244 (22.8)	302 (20.8)	207 (18.1)	219 (19.0)	0.08
Lung Disease	703 (14.0)	186 (17.2)	232 (15.7)	135 (11.4)	150 (12.7)	<0.001
Dementia	127 (2.1)	66 (6.0)	35 (2.2)	17 (1.1)	9 (0.6)	<0.001
Cancer	1272 (25.7)	280 (25.8)	388 (24.6)	291 (25.5)	313 (26.8)	0.70
Multimorbidity	3554 (68.8)	915 (79.8)	1130 (72.6)	797 (68.9)	712 (58.7)	<0.001
Number of Hospitalizations	1.54 (0.04)	1.64 (0.08)	1.55 (0.07)	1.58 (0.14)	1.35 (0.06)	1.00
Cardiometabolic Dysfunction<br>Elevated Waist Circumference	3014 (63.1)	784 (68.8)	902 (68.3)	696 (64.4)	632 (54.3)	<0.001
Diabetes	1174 (22.4)	353 (31.5)	414 (26.8)	237 (21.1)	170 (14.0)	<0.001
High Blood Pressure	3274 (62.8)	818 (70.8)	1050 (66.6)	745 (63.5)	661 (53.9)	<0.001
Metabolic Syndrome Score	1.46 (0.02)	1.70 (0.04)	1.54 (0.03)	1.48 (0.03)	1.22 (0.04)	0.008
Cardiovascular Disease<br>Myocardial Infarction	683 (12.5)	201 (17.3)	215 (13.4)	152 (12.3)	115 (9.1)	<0.001
Stroke	488 (8.4)	178 (15.3)	181 (10.6)	72 (6.1)	57 (4.1)	<0.001
Other Frailty Domain<br>Unintentional Weight Loss	636 (11.0)	215 (18.0)	221 (13.2)	103 (8.1)	97 (7.1)	<0.001
Low Physical Activity	1381 (25.2)	448 (38.4)	506 (32.0)	240 (20.2)	187 (14.8)	<0.001
Fatigue	1299 (25.7)	406 (37.9)	476 (32.5)	234 (21.5)	183 (15.4)	<0.001
Baseline Grip Strength	27.99 (0.22)	23.79 (0.55)	26.48 (0.37)	28.88 (0.40)	30.89 (0.37)	0.001
Yearly Change in Grip Strength	−0.60 (0.04)	−0.80 (0.11)	−0.68 (0.07)	−0.54 (0.09)	−0.46 (0.06)	1.00
Biological Measures<br>Hemoglobin A1c	5.63 (0.02)	5.71 (0.03)	5.66 (0.03)	5.66 (0.03)	5.55 (0.02)	1.00
CRP	1.82 (0.03)	2.07 (0.09)	1.91 (0.07)	1.85 (0.06)	1.65 (0.05)	0.90
IL6	6.45 (0.31)	6.38 (0.54)	6.13 (0.31)	6.64 (0.50)	6.58 (0.63)	1.00

Table S3. Baseline characteristics of demographic information for older adults stratified by baseline grip strength quartiles among 2011 cohort.

Variable	Total (N = 4961) N (%)	Q1 (N = 1107) N (%)	Q2 (N = 1607) N (%)	Q3 (N = 1116) N (%)	Q4 (N = 1131) N (%)	*p*-Value
Yearly Change in Grip Strength	−0.60 (0.04)	0.20 (0.08)	−0.51 (0.08)	−0.74 (0.07)	−1.15 (0.07)	<0.001
Demographics<br>Age<br>65–69 years	1026 (30.3)	85 (12.4)	239 (22.8)	274 (34.4)	428 (48.0)	<0.001
70–74 years	1114 (26.2)	147 (17.6)	331 (25.4)	286 (28.3)	350 (31.2)	
75–79 years	1043 (19.4)	233 (23.9)	352 (21.4)	250 (19.9)	208 (13.6)	
80–84 years	956 (13.8)	267 (21.3)	379 (17.8)	209 (12.4)	101 (5.3)	
85–89 years	822 (10.3)	375 (24.8)	306 (12.6)	97 (5.0)	44 (2.0)	
Gender<br>Male	2139 (44.6)	493 (44.9)	654 (41.9)	489 (44.7)	503 (47.5)	0.05
Female	2822 (55.4)	614 (55.1)	953 (58.1)	627 (55.3)	628 (52.5)	
Race<br>White	3553 (82.8)	790 (78.0)	1131 (80.7)	799 (84.1)	833 (87.6)	<0.001
Black	980 (7.3)	185 (6.5)	310 (7.2)	237 (7.8)	248 (7.5)	
Hispanic	269 (6.1)	92 (10.2)	99 (7.2)	53 (5.3)	25 (2.7)	
Other	159 (3.8)	40 (5.2)	67 (4.9)	27 (2.8)	25 (2.2)	
Education<br><High School	1149 (18.7)	352 (29.4)	394 (20.2)	214 (15.0)	189 (12.7)	<0.001
High School	1344 (26.7)	308 (27.7)	422 (26.6)	326 (29.5)	288 (23.7)	
Some College	663 (14.4)	116 (11.2)	216 (14.2)	170 (15.7)	161 (15.7)	
≥College Degree	1782 (40.2)	331 (31.7)	555 (39.0)	405 (39.8)	491 (47.9)	
Marital Status<br>Married	2518 (56.8)	477 (47.2)	771 (53.3)	598 (59.0)	672 (65.8)	<0.001
Separated/Divorced	608 (12.2)	127 (11.6)	187 (12.2)	139 (12.5)	155 (12.4)	
Widowed	1547 (25.2)	437 (35.3)	564 (29.4)	307 (22.1)	239 (15.9)	
Never Married	175 (3.3)	44 (3.7)	61 (3.9)	40 (3.2)	30 (2.5)	
Living with Partner	108 (2.5)	21 (2.2)	21 (1.2)	32 (3.2)	34 (3.4)	
Residence Type<br>Community	4762 (96.1)	1037 (93.4)	1528 (94.7)	1079 (97.1)	1118 (98.8)	<0.001
Residential Care	199 (3.9)	70 (6.6)	79 (5.3)	37 (2.9)	13 (1.2)	
Income<br>USD 0–24,999	1314 (23.5)	348 (30.2)	456 (26.4)	287 (22.5)	223 (16.3)	<0.001
USD 25,000–49,999	718 (14.8)	142 (13.3)	206 (13.0)	183 (16.9)	187 (16.0)	
USD 50,000–74,999	411 (9.3)	77 (7.1)	132 (9.6)	96 (9.7)	106 (10.4)	
USD 75,000–99,999	211 (5.4)	34 (3.9)	59 (4.6)	47 (5.3)	71 (7.4)	
USD 100,000–199,999	219 (5.8)	31 (3.2)	52 (4.3)	50 (5.4)	86 (9.8)	
USD 200,000+	77 (2.1)	10 (1.1)	17 (1.3)	23 (3.0)	27 (3.0)	
MISSING	2011 (39.0)	465 (41.1)	685 (40.8)	430 (37.2)	431 (37.2)	
Language<br>Spanish	717 (16.4)	202 (21.0)	221 (16.4)	146 (14.8)	148 (14.9)	0.005
English	4091 (83.6)	851 (79.0)	1320 (83.6)	950 (85.2)	970 (85.1)	
Insurance<br>Medicaid	610 (9.5)	176 (15.0)	224 (11.4)	110 (6.7)	100 (6.1)	<0.001
Medigap	2689 (56.7)	578 (52.5)	856 (55.4)	632 (60.0)	623 (58.2)	
Tricare	156 (3.3)	24 (2.1)	53 (3.4)	42 (4.4)	37 (3.1)	
Medicare only	1506 (30.4)	329 (30.3)	474 (29.8)	332 (28.9)	371 (32.6)	
Health<br>Current Smoker	381 (15.3)	72 (16.8)	114 (15.1)	85 (12.5)	110 (17.3)	0.12

Comorbidities<br>Arthritis	2630 (51.5)	655 (59.1)	959 (58.6)	537 (48.2)	479 (40.8)	<0.001
Osteoporosis	972 (20.0)	237 (22.2)	373 (23.6)	205 (20.0)	157 (14.2)	<0.001
Lung Disease	703 (14.0)	165 (15.3)	254 (15.7)	138 (12.0)	146 (13.0)	0.04
Dementia	127 (2.1)	57 (4.7)	49 (3.0)	12 (0.5)	9 (0.7)	<0.001
Cancer	1272 (25.7)	276 (25.3)	443 (27.4)	265 (23.4)	288 (26.1)	0.23
Multimorbidity	3554 (68.8)	856 (77.3)	1235 (74.4)	739 (63.8)	724 (60.9)	<0.001
Number of Hospitalizations	1.54 (0.04)	1.65 (0.07)	1.55 (0.06)	1.47 (0.09)	1.44 (0.12)	1.00
Cardiometabolic Dysfunction<br>Elevated Waist Circumference	3014 (63.1)	700 (64.6)	916 (62.8)	690 (62.3)	708 (63.2)	0.79
Diabetes	1174 (22.4)	303 (28.3)	411 (25.0)	248 (21.4)	212 (16.1)	<0.001
High Blood Pressure	3274 (62.8)	742 (66.7)	1101 (65.8)	711 (60.4)	720 (58.8)	<0.001
Metabolic Syndrome Score	1.46 (0.02)	1.59 (0.04)	1.47 (0.02)	1.43 (0.03)	1.37 (0.03)	0.05
Cardiovascular Disease<br>Myocardial Infarction	683 (12.5)	183 (15.8)	247 (14.1)	139 (11.0)	114 (9.7)	<0.001
Stroke	488 (8.4)	150 (13.1)	186 (9.9)	95 (7.5)	57 (4.1)	<0.001
Other Frailty Domain<br>Unintentional Weight Loss	636 (11.0)	192 (16.2)	213 (11.6)	129 (10.3)	102 (7.1)	<0.001
Low Physical Activity	1381 (25.2)	397 (35.2)	484 (27.8)	263 (21.3)	237 (18.4)	<0.001
Fatigue	1299 (25.7)	353 (32.2)	496 (30.9)	252 (22.6)	198 (17.7)	<0.001
Baseline Gait Speed	0.86 (0.01)	0.71 (0.01)	0.81 (0.01)	0.90 (0.01)	0.97 (0.01)	<0.001
Yearly Change in Gait Speed	−0.03 (0.00)	−0.04 (0.00)	−0.03 (0.00)	−0.02 (0.00)	−0.02 (0.00)	0.12
Biological Measures<br>Hemoglobin A1c	5.63 (0.02)	5.68 (0.05)	5.65 (0.02)	5.64 (0.03)	5.58 (0.03)	1.00
CRP	1.82 (0.03)	2.12 (0.07)	1.85 (0.05)	1.79 (0.06)	1.69 (0.05)	0.02
IL6	6.45 (0.31)	6.76 (0.56)	5.94 (0.33)	6.43 (0.56)	6.86 (0.66)	1.00
